# The effects of thoracic surgery techniques on postoperative pain and comparison of intercostal interventions

**DOI:** 10.1097/MD.0000000000048555

**Published:** 2026-05-08

**Authors:** Onur Bayrakçi

**Affiliations:** a Department of Thoracic Surgery, Gaziantep City Hospital, Gaziantep, Turkey.

**Keywords:** biportal, pain, uniportal, VAS, VATS

## Abstract

Surgical treatments of the thorax are one of the most painful surgeries known. Postoperative pain is an important factor affecting treatment success, hospital stay, complications, duration of chest tube placement, and respiratory dynamics. The aim of this study was to compare the effects of analgesic treatments on postoperative pain in patients who underwent surgery with uniportal or biportal VATS (video-assisted thoracoscopic surgery), minithoracotomy, and thoracotomy. This retrospective, single-center observational analytical study was conducted from October 7, 2023, to June 25, 2024, in Department of Thoracic Surgery at Gaziantep City Hospital. Patients who were over 18 years of age, had undergone thoracic surgery were included in the study. Demographic data, comorbidities, surgical techniques, postoperative pain severity according to VAS (visual analogue scale) score, analgesic treatments and interventions, duration of chest tube placement, complications, hospital length of stay were reviewed. A total of 319 patients were examined in the study retrospectively. The gender distribution of the patients were 70.22% male, 29.78% female. Postoperative pain VAS values were 0 to 2 very mild 36.2%, 3 to 4 mild 26.3%, 5 to 6 moderate 27.8%, 7 to 8 severe 11.1%, 9 to 10 very severe 6.2% in uniportal VATS. Pain VAS values for biportal VATS were found as 0 to 2 36.2%, 3 to 4 26.3%, 5 to 6 21.1%, 7 to 8 15.5%, and 9 to 10 18.8%. The VAS values of pain in minithoracotomy were 0 to 2 24.7%, 3 to 4 37.3%, 5 to 6 33.4%, 7 to 8 26.7%, and 9 to 10 18.8%. Postoperative pain VAS values were 0 to 2 very mild 2.9%, 3 to 4 mild 10.1%, 5 to 6 moderate 17.7%, 7 to 8 severe 46.7%, 9 to 10 very severe 56.2% in thoracotomy. Very mild pain value on visual analog scale was related with uniportal VATS, biportal VATS, and minithoracotomy. Very severe pain was only associated with thoracotomy. There was no significant difference in pain between uniportal VATS and biportal. Thoracoscopic surgical techniques (uniportal and biportal VATS) were related with less pain than minithoracotomy and thoracotomy. Intercostal block with uniportal VATS was also associated with less pain than other interventions. Intercostal crush and intercostal ligation were related with lower pain than other interventions in all surgical techniques. No statistically significant difference was found between intercostal crush and ligation on low pain values of visual analog scale.

## 1. Introduction

Surgical treatments of the thorax are one of the most painful surgeries known. The mechanism of pain transmission varies and is related to patient factors as well as surgical technique.^[1]^ Causes of pain in thoracic surgeries may include mechanical injury, compression or injury of intercostal nerves, and placement of a thoracic tube.^[2]^ Postoperative pain is an important factor affecting treatment success, hospital stay, complications, duration of chest tube placement, and respiratory dynamics. The incidence of pain is known to be between 30% to 50%,^[3]^ but rates of up to 75% have been reported in the literature and, the incidence of severe pain reaches 10%.^[4]^ Pain that recurs and persists along the incision for at least 2 months after surgery is defined as chronic. Chronic pain is associated with decreased activity and poor quality of life in the patient. The incidence of chronic numbness and chronic pain is 30%.^[5,6]^

Minimally invasive thoracic surgery has become increasingly popular in recent years. It has a place in the treatment of many diseases such as pneumothorax, pleural effusion, solitary pulmonary nodule, and lung cancer. Video-assisted thoracoscopic surgery (VATS), a minimally invasive technique in thoracic pathologies, has been shown to be safe and effective.^[7,8]^ The intrathoracic cavity is reached with an approximately 1 to 2 cm incision in the intercostal space. These incisions are used as ports for optical and surgical materials. In the VATS technique, surgical procedures can be performed with uniportal, biportal, triportal or more ports.^[9–11]^ The main point in VATS is not to use a retractor that will widen the intercostal space. In surgery, maximal exploration and controlled procedures performed on the tissues in the thoracic area are essential. Switching from multiportal VATS technique to biportal VATS technique is possible only with surgical experience and adequate exploration of pathological findings.^[12]^ Considering the number of incisions, uniportal VATS is of course the most minimally invasive intervention technique. Minithoracotomy technique is another technique in which thoracic and cardiovascular surgical procedures can be performed with anterior, lateral, posterolateral, and posterior approaches.^[13]^ A minithoracotomy is slightly larger (5–8 cm) than a VATS incision and slightly smaller than a thoracotomy. Retractors are not used in VATS, but are used in thoracotomies. Thoracotomy is usually performed via a posterolateral incision to access the hilar structures. The incision size may vary between surgeons, usually measuring >8 to 10 cm.

Effective pain management is important to ensure patient comfort, accelerate recovery, prevent complications, and improve overall patient outcomes in thoracic surgery.^[14,15]^ Therefore, it is necessary to evaluate the patient’s pain comprehensively and determine the need for treatment. The visual analog scale (VAS) is defined as a practical pain assessment scale. With this scale, the patient’s pain can be evaluated numerically, from no pain at all to the most severe and unbearable pain.^[16,17]^ The degree of severity of pain can be described and analgesic treatments are done accordingly.

The aim of this study was to compare the effects of analgesic treatments such as intercostal blockade, crush, and ligation on postoperative pain in patients who underwent surgery with uniportal or biportal VATS, minithoracotomy, and thoracotomy.

## 2. Methods

### 2.1. Study design and study population

This retrospective, single-center observational analytical study was conducted from October 7, 2023, to June 25, 2024, in Department of Thoracic Surgery at Gaziantep City Hospital. The Institutional Research Ethics Committee approved the study protocol (number 34/2024).

The files were reviewed of all patients who admitted to the Department of Thoracic Surgery and continued their postoperative follow-up (n = 452). Patients who were over 18 years of age, had undergone thoracic surgery, and had complete access to all data required for the study were included in the study (n = 319). Patients who had not undergone thoracic surgery, whose data were missing or inaccessible, and who were younger than 18 years of age were excluded from the study.

### 2.2. Outcomes

The aim of this study was to compare the effects of analgesic treatments with uniportal or biportal VATS, minithoracotomy, and thoracotomy. Pain is one of the most important problems after thoracic surgeries. The effects of analgesic drugs, intercostal blockade, intercostal mass crushing, and intercostal nerve ligation on postoperative pain were studied. According to the VAS scale, pain intensities were determined in thoracic surgical methods such as uniportal or biportal VATS, minithoracotomy or thoracotomy. In respect of surgical techniques, which of the methods applied for pain control was more effective was determined.

### 2.3. Clinical data collection

Demographic data, comorbidities, smoking or alcohol use, surgical techniques done (uniportal VATS, biportal VATS, minithoracotomy, and thoracotomy), Postoperative pain severity according to VAS score, analgesic treatments, and interventions (medical drugs, intercostal blockade, intercostal mass crush, and intercostal ligation), thoracic tube length of stay, complications, intensive care and hospital length of stay, and various laboratory tests (including C-reactive protein [CRP], white blood cells, lymphocytes, neutrophils, procalcitonin, D-dimer (DD), lactate dehydrogenase [LDH], and creatine) were reviewed prospectively.

### 2.4. Determination of pain intensity and protocol of analgesic interventions

**Figure 1.**
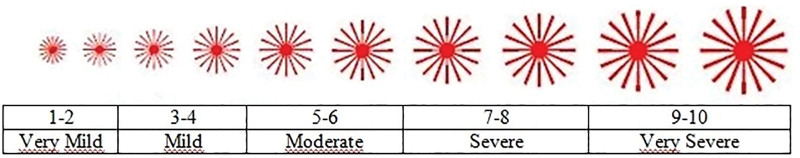
Pain intensity values on the visual analog scale.

The severity of pain should be determined to monitor the treatment in the postoperative period in thoracic surgeries. VAS was used in the study.^[17]^ Accordingly, the severity of pain was scored between 0 and 10. Pain intensity was expressed 1 to 2 as very mild, 3 to 4 as mild, 5 to 6 as moderate, 7 to 8 as severe, 9 to 10 as very severe and unbearable (Fig. 1).

**Figure 2.**
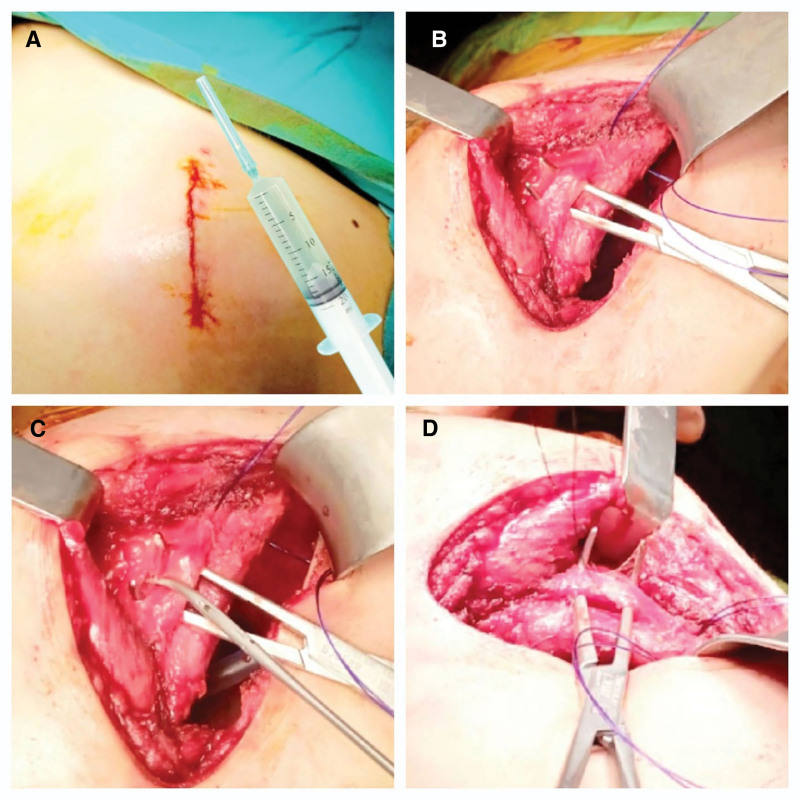
Image of intercostal interventions. (A) Intercostal nerve block. (B) Appearance of intercostal mass. (C) Crushing of the intercostal mass. (D) Intercostal mass ligation.

In the postoperative period, all patients admitted to the intensive-care unit or thoracic surgery ward received medical analgesic treatment. Medical analgesic drugs were tramadol hydrochloride 200 mg/day and paracetamol 2000 mg/day. Immediately after the surgery, intercostal blockade was done with a total of 400 mg of prilocaine hydrochloride at 2 levels from the posterior hemithorax. Intercostal mass was crushed by clamping the intercostal muscle, nerve, and vascular bundle at the same level as the surgical procedure. Intercostal ligation was done by exploring and suturing the intercostal nerve at the same level as the surgery (Fig. 2).

**Figure 3.**
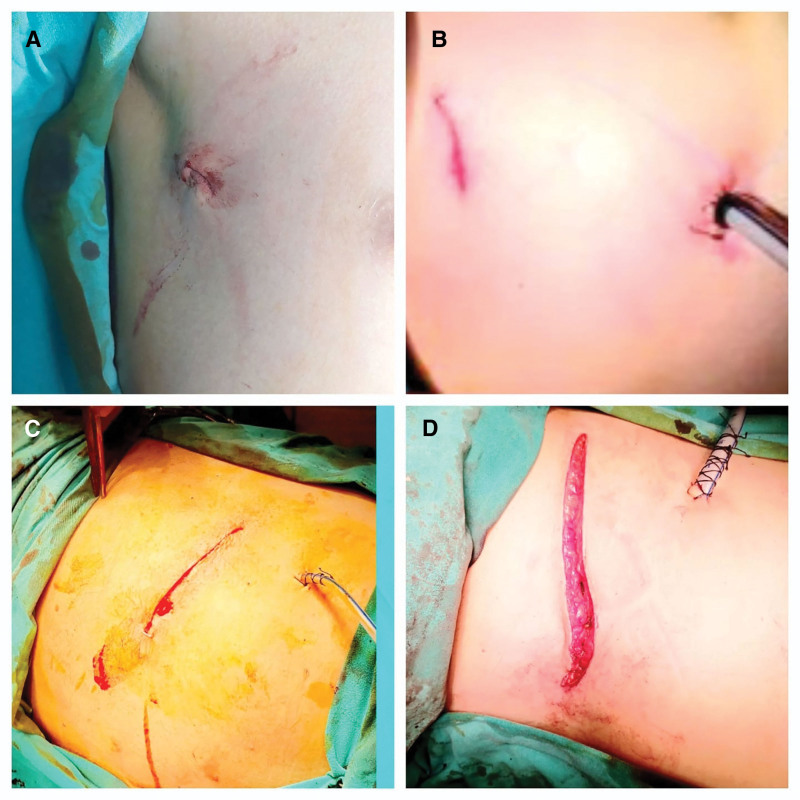
Techniques of thoracic surgery. (A) Image of uniportal VATS incision. (B) Image of biportal VATS incision. (C) Image of minithoracotomy incision. (D) Image of thoracotomy incision. VATS = video-assisted thoracoscopic surgery.

Surgical techniques were VATS (uniportal or biportal), minithoracotomy, and thoracotomy. VATS was usually performed with an approximately 2 to 3 cm incision in the 4th intercostal space in the anterior axillary line (uniportal). During exploration, a second port was opened in the 6th intercostal space in the middle or posterior axillary line, as needed (biportal). Minithoracotomy was often performed via the 5th intercostal space with an approximately 6 to 8 cm incision lateral to the hemithorax. Thoracotomy was done as a standard posterolateral 12 to 16 cm incision at the 5th or 6th intercostal spaces^[18]^ (Fig. 3).

### 2.5. Statistical analysis

The adequacy of the total population and sample numbers of the groups were determined by G-power analysis for scientific significance. The homogeneity of the population distribution was assessed with the Kolmogorov–Smirnov test. The effects of surgical techniques and intercostal interventions on pain were examined by both univariate and multivariate analyses. Comparisons between groups were made with the Mann–Whitney *U* test or the Chi-squared test. The Kruskal–Wallis test was used for multiple dependent variables. Results were reported as mean ± SD, median, number (n), and percentage (%), with *P*-value <.05 considered significant. Analyses were performed using SPSS v22.0.

## 3. Results

A total of 319 patients were examined in the study retrospectively. The gender distribution of the patients were 70.22% male, 29.78% female. The proportion of patients without comorbidity was 48.90% and patients with comorbidity was 51.10% (diabetes mellitus 26.02%, hypertension 25.02%, chronic obstructive pulmonary disease 6.90%, respectively). Smoking was 76.49% and alcohol use was 16.30% (Table 1). The mean values of laboratory parameters were white blood cells 9900/µL, NEU 7790/µL, lymphocytes 910/µL, CRP 132.4 mg/L, 2.22 µg/L, LDH 551.5 U/L, DD 8.60 mg/L, and creatine 1.36 mg/dL. There was no statistically significant relationship comorbidities (*P:* .1044) and laboratory parameters (CRP *P:* .962, LDH *P*: .1034, and DD *P*: .987) between VAS pain value as an independent variable (Table 2). The groups that used only painkillers and those that underwent intercostal intervention (interkostal blok, crushing, and ligation) along with painkillers were comparatively examined according to surgical techniques.

**Table 1:** Demographic data of the study.

Parameters	n	Mean	Standard deviation
Ages	319	55.4 (17–81)	20.2
Gender			
Male	224	70.22%	
Female	95	29.78%	
Comorbidity			
Yes	163	51.10%	
Hypertension	80	25.08%	
Diabetes mellitus	83	26.02%	
Chronic obstructive pulmonary disease	22	6.90%	
No		48.90%	
Habitude			
Use of cigarettes	244	76.49%	
Use of alcohol	52	16.30%	

**Table 2:** Laboratory data of the study.

Laboratory parameters	Mean	Ranges (min–max)	References	SD	95% (±)
White blood cells	9900	1790–36500	4500–11,000 mcL	575.52	0.634
Neutrophil	7790	800–28,400	1500–7000 mcL	5.08	0.568
Lymphocyte	910	110–12,300	1000–4800 mcL	0.85	0.655
C-reactive protein	132.4	5.4–365	10–100 mg/L	83.5	18.82
Procalcitonin	2.22	0.02–100	0–0.05 µg/L	10.58	3.080
Lactate dehydrogenase	551.5	2.73–1520	125–220 U/L	240.3	26.20
D-Dimer	8.60	0.23–45.9	0–0.50 mg/L	42.3	0.735
Creatine	1.36	0.32–9.69	0.5–1.2 mg/dL	1.38	0.158

According to their localizations, the mean pain VAS of right surgical procedures was 4.319 and of left surgical procedures was 4.410. There was no statistically significant difference according to localizations (*P*: 1.124). According to surgical techniques, the mean VAS pain scores were 3.827 in uniportal VATS, 3.753 in biportal VATS, 4.271 in minithoracotomy, and 5.923 in thoracotomy. There is no statistically significant difference between uniportal VATS and biportal VATS (*P*: .962) and between minithoracotomy and thoracotomy (*P*: .811) in terms of postoperative pain according to VAS values. Minimally invasive thoracoscopic surgical procedures (uniportal and biportal VATS) are statistically significantly less postoperatively painful than both minithoracotomy (*P*: .0012 and *P*: .0024) and thoracotomy (*P*: .0001 and *P*: .0003), respectively.

Postoperative pain VAS values were 0 to 2 very mild 36.2%, 3 to 4 mild 26.3%, 5 to 6 moderate 27.8%, 7 to 8 severe 11.1%, 9 to 10 very severe 6.2% in uniportal VATS. Pain VAS values for biportal VATS were found as 0 to 2 36.2%, 3 to 4 26.3%, 5 to 6 21.1%, 7 to 8 15.5%, and 9 to 10 18.8%. The VAS values of pain in minithoracotomy were 0 to 2 24.7%, 3 to 4 37.3%, 5 to 6 33.4%, 7 to 8 26.7%, and 9 to 10 18.8% (Table 3). Postoperative pain VAS values were 0 to 2 very mild 2.9%, 3 to 4 mild 10.1%, 5 to 6 moderate 17.7%, 7 to 8 severe 46.7%, 9 to 10 very severe 56.2% in thoracotomy. Very mild VAS pain value was statistically associated (uniportal VATS *P*: .0034, biportal VATS *P*: .0241 minithoracotomy *P*: .0402). Mild VAS pain value was statistically associated (uniportal VATS *P*: .0101, biportal VATS *P*: .0203, minithoracotomy *P*: .0501, and thoracotomy *P*: .0481). Moderate VAS pain value was statistically related (uniportal VATS *P*: .0406, biportal VATS *P*: .0408, minithoracotomy *P*: .0385, and thoracotomy *P*: .0392). Severe VAS pain value was statistically related (uniportal VATS *P*: .0508, biportal VATS *P*: .0502, minithoracotomy *P*: .0203, and thoracotomy *P*: .0165). Very severe VAS pain value was statistically related (only thoracotomy *P*: .0061). There was no statistically significant difference between uniportal VATS and biportal VATS (*P*: .686), and between biportal VATS and minithoracotomy (*P*: .851), in terms of reducing VAS pain values in multivariate analysis. Uniportal VATS was statistically significant with lower pain values than both minithoracotomy (*P*: .0012) and thoracotomy (*P*: .0001). Biportal VATS was statistically significant with lower pain values than both minithoracotomy (*P*: .0026) and thoracotomy (*P*: .0003). Minithoracotomy (*P*: .0052) was statistically significant with lower pain values than thoracotomy in multivariate analysis (Table 4).

**Table 3:** VAS pain intensities according to surgical techniques.

VAS scale value of pain	Totally (N)	Uniportal VATS	Biportal VATS	Minithoracotomy	Thoracotomy
0 to 2 (very mild)	69	25 (36.2%)	25 (36.2%)	17 (24.7%)	2 (2.9%)
3 to 4 (mild)	99	26 (26.3%)	26 (26.3%)	37 (37.3%)	10 (10.1%)
5 to 6 (moderate)	90	25 (27.8%)	19 (21.1%)	30 (33.4%)	16 (17.7%)
7 to 8 (severe)	45	5 (11.1%)	7 (15.5%)	12 (26.7%)	21 (46.7%)
9 to 10 (very severe)	16	1 (6.2%)	3 (18.8%)	3 (18.8%)	9 (56.2%)

VAS = visual analog scale; VATS = video-assisted thoracoscopic surgery.

**Table 4:** Statistical analysis of intercostal interventions according to surgical techniques.

Parameters	n	Uniportal VATS		Biportal VATS		Minithoracotomy		Thoracotomy	
		n	*P*	n	*P*	n	*P*	n	*P*
Analgesic treatment	77	19	.2495	18	.8414	24	.3757	16	.4531
Intercostal block	91	29	.0032	19	.4301	23	.1693	20	.0791
Intercostal crush	96	21	.0273	29	.0411	29	.0032	17	.0004
Intercostal ligation	55	12	.0502	11	.0562	20	.0417	12	.0820

VATS = video-assisted thoracoscopic surgery.

Although analgesic drugs for postoperative pain have relatively less pain VAS value in uniportal and biportal VATS techniques, it is not statistically significant in any technique (uniportal VATS *P*: .2495, biportal VATS *P*: .8414, minithoracotomy *P*: .3757, and thoracotomy *P*: .4531). Intercostal block was associated with uniportal VATS (*P*: .0032), and thoracotomy (at a low level of evidence *P*: .0791). In multivariate analysis, intercostal block application is statistically significant in the uniportal VATS technique (*P*: .0002) with lower VAS pain values compared to thoracotomy. The intercostal crush method was statistically associated with all surgical techniques (uniportal VATS *P*: .0273, biportal VATS *P*: .0411, minithoracotomy *P*: .0032, and thoracotomy *P*: .0004). In multivariate analysis, there is no statistically significant difference between uniportal VATS and biportal VATS on VAS pain values in intercostal crush (*P*: .0816). Both thoracoscopic techniques (uniportal and biportal VATS) were statistically significant in reducing VAS pain values with minithoracotomy (*P*: .0216 vs *P*: .0124) and thoracotomy (*P*: .0134 and *P*: .0034) separately in multivariate analysis. The intercostal ligation method was statistically related with all surgical techniques (low levels of evidence uniportal VATS *P*: .0502, biportal VATS *P*: .0562, minithoracotomy *P*: .0417, and thoracotomy *P*: .0820). In multivariate analysis, intercostal ligation was found to be statistically significant only for minithoracotomy technique in reducing VAS pain value (*P*: .0217) (Table 5).

**Table 5:** Average VAS pain values of surgical techniques.

Parameters	n	Average value of pain	Standard deviation	CI 95%
Technique of surgery	319	4.370	2.065	0.227
Uniportal VATS	81	3.827	1.815	0.401
Biportal VATS	77	3.753	1.954	0.444
Minithoracotomy	96	4.271	1.803	0.365
Thoracotomy	65	5.923	2.100	0.521
Localization of surgery				
Right	141	4.319	2.040	0.340
Left	178	4.410	2.090	0.309

VAS = visual analog scale, VATS = video-assisted thoracoscopic surgery.

Complication (most often pneumothorax) was found to be statistically significant in thoracotomy patients with intercostal block (*P*: .0492). The length of stay of the thorax tube was statistically significant only in thoracotomies in multivariate analysis (analgesic treatment *P*: .0453, intercostal block *P*: .0001, intercostals crush *P*: .0002, and intercostal ligation *P*: .0033). Admission to intensive-care unit was not statistically related with any surgical techniques or intercostal interventions. The length of hospital stay was statistically significant in patients who underwent analgesic treatment with uniportal VATS (*P*: .0413) technique and in patients who underwent intercostal ligation with thoracotomy (*P*: .0493) in multivariate analysis (Table 6).

**Table 6:** Multivariate statistical analysis of parameters.

Parameters	Analgesic treatment		Intercostal block		Intercostal crush		Intercostal ligation	
Complication	Mean	*P*	Mean	*P*	Mean	*P*	Mean	*P*
Uniportal VATS	0		0.069	.7039	0.047	.4347	0	
Biportal VATS	0.055	.4347	0.157	.4121	0		0	
Minithoracotomy	0		0.087	.1943	0		0	
Thoracotomy	0.125	.8254	0.300	.0492	0.176	.5241	0.250	.1364
*Length of stay of the thorax tube*								
Uniportal VATS	1.842	.1013	1.862	.0520	2.238	.7711	1.583	.1070
Biportal VATS	2.222	.6353	1.789	.0729	1.897	.1920	1.909	.7773
Minithoracotomy	2.500	.8086	2.435	.3501	2.241	.9766	2.300	.7417
Thoracotomy	3.188	.0453	3.550	.0001	3.412	.0002	3.250	.0033
*Length of stay of the ICU*								
Uniportal VATS	0.105	.5524	0.137	.3575	0.095	.3437	0.083	.4404
Biportal VATS	0.055	.9126	0.105	.2914	0.103	.0964	0.090	.2983
Minithoracotomy	0		0.217	.1643	0.069	.9767	0	
Thoracotomy	0.312	.6381	0.650	.9207	0.352	.4376	0.333	.5375
*Length of stay of the hospital*								
Uniportal VATS	2.789	.0413	3.862	.0932	4.238	.3432	2.667	.7515
Biportal VATS	3.222	.3176	3.789	.3902	3.897	.0964	3.000	.9145
Minithoracotomy	3.458	.9332	4.478	.2358	4.241	.9512	3.350	.0734
Thoracotomy	4.125	.0626	5.550	.0593	5.412	.0823	4.500	.0493

ICU = intensive care unit, VATS = video-assisted thoracoscopic surgery.

## 4. Discussion

Pain management in the postoperative period is important in terms of morbidity and patient satisfaction. Thoracic surgery involves anatomical structures such as the heart, lungs, trachea and esophagus, and pain is inevitable.^[16]^ It has been reported that VAS in pain management has not been sufficiently investigated in the field of thoracic surgery.^[19]^ Therefore, the effects of analgesic approaches on VAS values in thoracic surgery techniques were investigated in this study. VAS pain value was measured immediately after surgery and at 24 hours. The mean of the first postoperative measurements of all surgical procedures was 7.2, and the mean of the 24th hour measurements was 4.9. Uniportal VATS technique has been reported to reduce postthoracotomy pain syndrome.^[20]^ Similarly, in this study, VAS pain values of patients who underwent surgery with uniportal VATS were lower than other techniques. It has been stated that the uniportal VATS technique is more advantageous in terms of pain than multiportal VATS in a study.^[20]^ However, in this study, no statistically significant difference was found in terms of pain between uniportal VATS and biportal VATS. Of course, having 2 port incisions instead of one is a cosmetic disadvantage. Sometimes, exploration is inadequate during surgery, complications related to critical vascular structures may be potential, or an additional post incision is required in hilar region dissections. High postoperative pain may cause postoperative pneumonia, atelectasis, and prolonged healing process. Since no significant difference was found in terms of pain in thoracoscopic surgeries (uniportal and biportal VATS), it can be preferred if necessary. A study reported the advantage of minithoracotomy surgery due to less postoperative pain compared to sternotomy.^[21]^ Similarly, although minithoracotomy caused slightly more pain than thoracoscopic surgeries, it was more advantageous than thoracotomy in this study. In this respect, it supports the idea that a smaller incision may cause less pain.

Postoperative pain management has traditionally been known to be very important and has been noted in many studies as reported in one study.^[22]^ The surgeon of the operation naturally wants the patient to have no pain. Unfortunately, this is not possible due to tissue irritation or damage. Therefore, surgeons prefer pain medications based on literature support, traditional conventional approaches, or their own experience. Opioids, nonopioids, simple or combined analgesics, anti-inflammatory, antirheumatic, myorelaxant, etc can be used to prevent or minimize postoperative pain for the patient. Patients using tramadol hydrochloride and paracetamol were included in this study. Although postoperative pain had a minimal reducing effect on VAS values, it did not have statistically significant effects compared to other methods. The effects of local anesthetic agents such as prilocaine and lidocaine are known.^[23]^ Lidocaine and prilocaine are used for pain relief in suturing, dental procedures, and many surgical procedures. Local anesthetic agents have traditionally been the preferred application of many surgeons. In the study, prilocaine hydrochloride was performed to 2 intercostal levels posterior to the level of the incision. Although intercostal block reduces VAS pain values, it is statistically significant in uniportal VATS. In thoracotomy with intercostal block, significance was found in reducing VAS pain value with low level of evidence. However, biportal VATS and minithoracotomy were not statistically significant. Intercostal radiofrequency ablation or cooled radiofrequency ablation treatments are performed for pain control.^[24]^ These methods aim to eliminate the feeling of pain by damaging the intercostal nerve through ablation. Preventing severe postoperative pain directly affects the treatment process and contributes to the stabilization of respiratory dynamics with patient satisfaction. Intercostal nerves are indirectly affected during anatomical reclosure of the operation area. In thoracic surgeries, the intercostal nerve at the level where the procedure is performed is already explored. In this study, intercostal crush and intercostal ligation are statistically significant in reducing VAS pain values in all surgical techniques. The absence of postoperative pain or its reduction to an acceptable level directly affects respiratory outcomes. Less pain, shorter hospital stay, earlier hospital discharge and patient satisfaction are positive results. Since both intercostal crush and intercostal ligation no difference neural symptoms with other techniques and were reversible procedures, no complications were observed at the end of the recovery process.

Tube thoracostomy is performed to provide drainage of air, blood or fluid after thoracic surgery operations. The short duration of the chest tube stay is ideal.^[25]^ This long period directly prolongs the duration of hospital stay. Longer length of stay of chest tube and longer hospital stay were associated with all intercostal interventions in thoracotomy. Admission to Intensive-care units was not statistically related with any technique or any intercostal interventions. Intercostal block, crush, and ligation were not statistically associated with the length of stay of the chest tube.

Limitations; the sample size of the study was statistically more than the minimum sample size required (G-power analysis). A much larger study population could have increased the level of evidence for the hypothesis. Additionally, pain thresholds can vary from person to person. If an individual’s pain threshold, or sensitivity to pain, could be determined in degrees, more specific results could be obtained. This would, of course, require additional research.

## 5. Conclusions

Very mild pain value on visual analog scale was related with uniportal VATS, biportal VATS, and minithoracotomy. Very severe pain was only associated with thoracotomy. There was no significant difference in pain between uniportal VATS and biportal. Thoracoscopic surgical techniques (uniportal and biportal VATS) were related with less pain than minithoracotomy and thoracotomy. Intercostal block with uniportal VATS was also associated with less pain than other interventions. Intercostal crush and intercostal ligation were related with lower pain than other interventions in all surgical techniques. No statistically significant difference was found between intercostal crush and ligation on low pain values of visual analog scale.

## Author contributions

**Conceptualization:** Onur Bayrakçi.

**Formal analysis:** Onur Bayrakçi.

**Investigation:** Onur Bayrakçi.

**Methodology:** Onur Bayrakçi.

**Project administration:** Onur Bayrakçi.

**Resources:** Onur Bayrakçi.

**Software:** Onur Bayrakçi.

**Validation:** Onur Bayrakçi.

**Visualization:** Onur Bayrakçi.

**Writing – original draft:** Onur Bayrakçi.

**Writing – review & editing:** Onur Bayrakçi.
